# Nutritional Modulation of AMPK-Impact upon Metabolic-Inflammation

**DOI:** 10.3390/ijms19103092

**Published:** 2018-10-09

**Authors:** Claire L. Lyons, Helen M. Roche

**Affiliations:** 1 Unit of Molecular Metabolism, Lund University Diabetes Center, Clinical Research Center, Lund University, 205 02 Malmö, Sweden; claire.lyons@med.lu.se; 2 Nutrigenomics Research Group, UCD Institute of Food and Health, UCD Conway Institute of Biomolecular and Biomedical Research, University College Dublin, Belfield, 4 Dublin, Ireland; 3 Institute of Global Food Security, Queen’s University Belfast BT7 1NN, Northern Ireland, UK

**Keywords:** AMPK, IL-1β, NLRP3, nutrition, dietary fatty acids, metabolic-inflammation, nutrigenomics

## Abstract

Nutritional status provides metabolic substrates to activate AMP-Activated Protein Kinase (AMPK), the energy sensor that regulates metabolism. Recent evidence has demonstrated that AMPK has wider functions with respect to regulating immune cell metabolism and function. One such example is the regulatory role that AMPK has on NLRP3-inlflammasome and IL-1β biology. This in turn can result in subsequent negative downstream effects on glucose, lipid and insulin metabolism. Nutrient stress in the form of obesity can impact AMPK and whole-body metabolism, leading to complications such as type 2 diabetes and cancer risk. There is a lack of data regarding the nature and extent that nutrient status has on AMPK and metabolic-inflammation. However, emerging work elucidates to a direct role of individual nutrients on AMPK and metabolic-inflammation, as a possible means of modulating AMPK activity. The posit being to use such nutritional agents to re-configure metabolic-inflammation towards more oxidative phosphorylation and promote the resolution of inflammation. The complex paradigm will be discussed within the context of if/how dietary components, nutrients including fatty acids and non-nutrient food components, such as resveratrol, berberine, curcumin and the flavonoid genistein, modulate AMPK dependent processes relating to inflammation and metabolism.

## 1. Introduction

**Figure 1:**
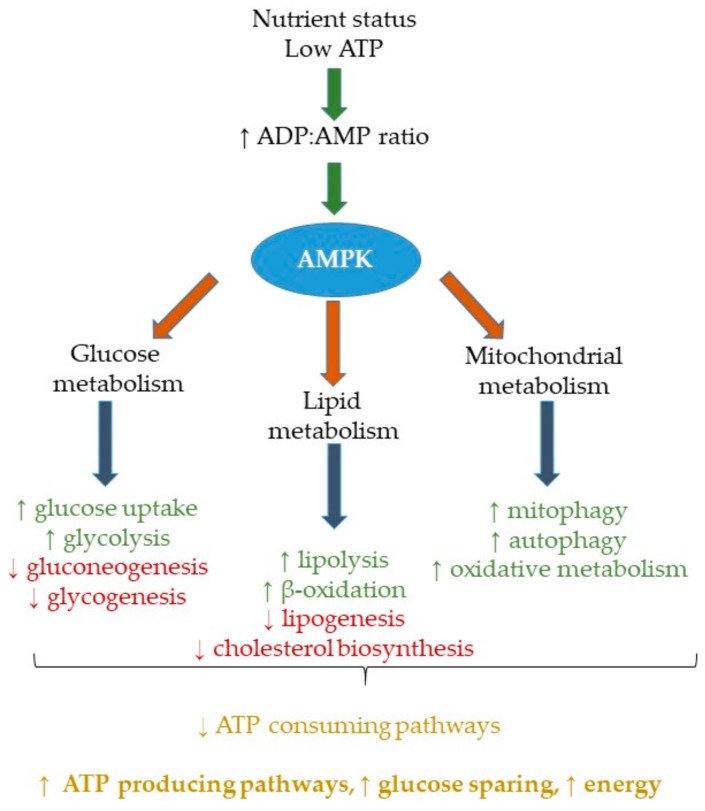
The role of AMP-Activated Protein Kinase (AMPK) on whole-body metabolism. AMPK is a nutrient sensor, which is activated in response to low adenosine triphosphate (ATP) levels, and an increased adenosine diphosphate: adenosine monophosphate (ADP:AMP) ratio. As a result, it activates pathways that produce ATP through glucose, lipid and mitochondrial metabolism pathways, thus increasing ATP levels. Conversely, pathways that deplete ATP are inhibited by AMPK. An ↑ arrow represents an upregulation of the process and ↓ represents a downregulation of the process. AMPK = AMP-Activated Protein Kinase, ATP = Adenosine triphosphate, ADP = Adenosine diphosphate, AMP = Adenosine monophosphate.

AMP-activated protein kinase (AMPK) is a serine/threonine kinase (once thought of as solely an energy sensor) and has since been assigned widespread roles in metabolism [1], inflammation [2] and Type 2 Diabetes (T2D) [3]. It is responsible for adapting cellular metabolism in response to nutritional and environmental variations. This involves activating pathways to produce energy whilst also inhibiting energy-consuming pathways. The body uses adenosine triphosphate (ATP) as an energy source, which is broken down to adenosine diphosphate (ADP) and then adenosine monophosphate (AMP). When ATP levels are low, the ADP:AMP ratio increases and in turn activates AMPK to activate pathways to replenish ATP levels and restore energy. AMPK is a heterotrimer composed of a catalytic α (α) (α1 and α2) subunit and regulatory beta (β) (β1 and β2) and γ (γ) (γ1, γ2 and γ3) subunits. AMPK can be activated through both allosteric and phosphorylation (p) means. The threonine-172 (Thr-172) residue within the activation loop of the kinase domain on the α-subunit is the main phosphorylation site for AMPK activation [4]. The upstream protein kinases, liver kinase B1 (LKB1), Ca^2+^/calmodulin-dependent protein kinase kinases (CaMKK) [5] and AMPK kinase (AMPKK) [4], are responsible for AMPK activation by phosphorylation. The allosteric activation of AMPK occurs through AMP and helps to prevent the dephosphorylation of AMPK [6]. AMP has also been shown to directly increase the CaMKKβ- and LKB1-mediated α-Thr^172^ phosphorylation [7]. AMPK activation, in response to low energy status, re-configures glucose, lipid and mitochondrial metabolism towards adenosine triphosphate (ATP) production and decreases anabolic pathways that would otherwise further deplete ATP levels (Figure 1). Insulin can inhibit AMPK activity through phosphorylation of the Ser^485/491^ in the α1/α2 site in multiple tissues, including skeletal muscle and liver, without affecting Thr^172^ phosphorylation, through the Akt pathway [8]. One of the main mechanisms of AMPK activation is the prevention of its dephosphorylation by AMP, especially within the context of metabolic disease [6]. The main pathways activated are those that are involved in growth and metabolism [9]. It activates fatty acid oxidation (FAO) to generate ATP and inhibits unnecessary pathways, such as fatty acid synthesis [1]. Alternatively, AMPK affects the cell cycle and neuronal membrane excitability, as a way to regulate ATP levels [10]. In this review, we will discuss how AMPK can be activated by nutrients, such as glucose and lipids, but is mainly determined by nutrient status. The widespread effects of AMPK on metabolism, ranging from nutrient to mitochondrial metabolism, will be covered. In times of stress, such as obesity or direct influence by fatty acids, AMPK activation becomes altered and leads to dysregulated metabolism. Furthermore, there is an increasing amount of research pointing to the effect of metabolic signals on immune function and inflammation, and how a reciprocal relationship exists. The literature to date has focused on the role of nutritional status on AMP activation and how this affects cellular processes and metabolism. However, whilst AMPK-mediated metabolic-inflammation is a critical biological interaction, there is a paucity of data in relation to the nature and the extent to which individual nutrients affects cellular process and metabolism, thus this will be a focus within the review.

## 2. AMPK Activation, Metabolism and Nutrient Status

Metabolism is fueled by the nutrients we consume and is a tightly regulated process. Nutrient status therefore has a direct effect on the energy status of the organism. On the cellular level, AMPK activation is dependent upon energy status in the form of low ATP levels, and an increased ADP:AMP ratio. It is activated by energetic stress characterized by low levels of ATP. AMPK activation re-configures cellular metabolism, switching on catabolic pathways to generate ATP and switching off anabolic pathways that would otherwise deplete ATP. The metabolic impact of AMPK activation/de-activation has already been thoroughly reviewed by Herzig and Shaw [11]. Briefly, as illustrated in Figure 1, lipid and glucose metabolism are re-configured to supply energy; whilst protein metabolism, particularly protein synthesis is shut down. AMPK activation promotes glucose uptake and glycolysis, and activates lipolysis and oxidation, which is associated with significant upregulation of mitochondrial metabolism, mitophagy and autophagy. Conversely, gluconeogenesis and glycogenesis, as well as fatty acid synthesis/lipogenesis and cholesterol biosynthesis, are attenuated. In essence, AMPK activation promotes glucose sparing and oxidative metabolism to generate maximal ATP from cellular energy substrates. This process is used by most quiescent cells, as opposed to the glucose dependent, glycolytic metabolism relied upon by activated immune and proliferating cells. The current review will focus on the effects of AMPK on cellular metabolism in the context of inflammation and whole-body responses to obesity. For more detailed reviews about AMPK and the effects on lipid, glucose and mitochondrial metabolism, the readers are directed to the following reviews [9,12,13]. Recent work suggests that glucose metabolites mediate AMPK activation via non-canonical energy sensing mechanisms. Whilst it is well acknowledged that cellular glucose status activates AMPK as described above, more recent work suggests that glucose status affects AMPK activation, independent of increasing AMP:ATP and ADP ratios. It was initially presumed that low glucose status activated AMPK via reduced glucose catabolism leading to ATP depletion via the canonical energy sensing mechanism. But Zhang and colleagues recently showed that AMPK was activated when mouse embryonic fibroblasts were transferred from high to low (below 5 mM) glucose concentrations without any changes in cellular AMP/ATP or ADP/ATP ratios [14]. It was demonstrated that during glycolysis, glucose is converted to fructose-1, 6-bisphosphate (FBP), which is then processed or sensed by FBP aldolases. Such low glucose status reduces FBP-bound aldolase, which in turn activates AMPK via LKB1 phosphorylation. Thus, metabolites such as FBP indicative of poor glucose availability modulate FBP aldolases, which in turn sense low FBP and activate AMPK.

### 2.1. Nutrient Status and Impaired AMPK Action

**Figure 2:**
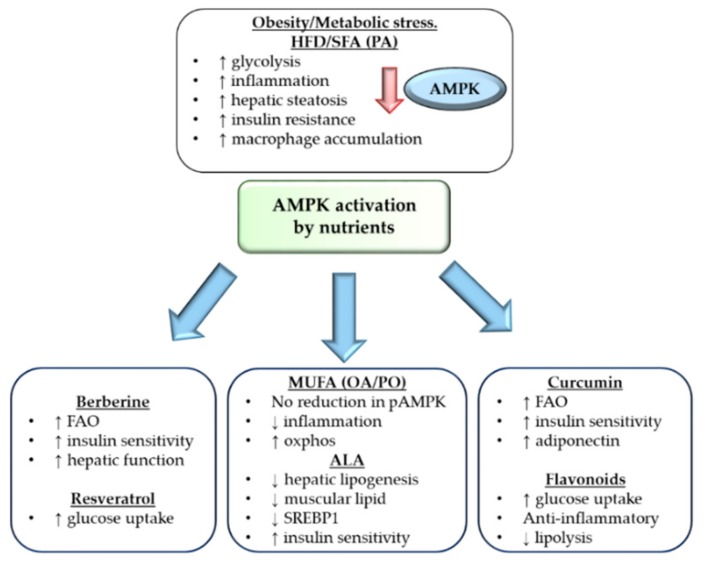
Whole body AMPK modulation and the impact of different nutrients on AMPK activation. Excess nutrient consumption through high fat diet (HFD) and obesity can downregulate AMP-Activated Protein Kinase (AMPK) expression and cause dysregulated metabolism, inflammation and insulin resistance. Nutrients, including monounsaturated fatty acids (MUFA), α linoleic acid (ALA), berberine, resveratrol, curcumin and flavonoids can all activate AMPK and downstream positive effects in relation to improved mitochondrial metabolism, improved liver function and reduced inflammation. Therefore, modulation of AMPK through nutrient intervention can improve whole body metabolism. An ↑ arrow represents an upregulation of the process or increased expression and ↓ represents a downregulation of the process or decreased expression. AMPK = AMP-Activated Protein Kinase, MUFA = Monounsaturated fatty acids, OA = Oleic acid, PO = Palmitoleic acid, ALA = α linoleic acid, SREBP1 = Sterol regulatory element-binding protein 1c, FAO = fatty acid oxidation.

On the whole-body level, energy status also affects AMPK activation. Obesity, in its simplest form, is caused by a chronic energy imbalance, wherein caloric intake exceeds caloric expenditure. This represents a metabolic stress event and hence affects AMPK activation, as illustrated in Figure 2. Obesity is associated with reduced AMPK activation, concomitant with alterations in glycolysis, insulin sensitivity, hepatic lipid metabolism and inflammation. The AMP metabolic stress signal results in β-myristoylation, allowing AMPK membrane association and facilitating the phosphorylation required for activation [7]. In man AMPK activity, as determined by its phosphorylation state, the P-AMPK/T-AMPK ratio, was reduced in visceral adipose tissue (VAT), rather than subcutaneous adipose tissue (SAT), of obese humans [15]. Obesity also affects immune cell AMPK status. Macrophage phosphorylated AMPK (pAMPK) expression was 33% lower in mouse models of genetic obesity compared to their lean counterparts [16]. Rats on a high fat diet (HFD) also show reduced renal AMPK activity [17]. Obesity arises as a result of excess energy intake, usually excess fat with or without surplus simple carbohydrates/sugars. Later in the review, we will deal with the impact of different dietary components within the context of obesity. It is important to note that high fat diets are usually preferentially enriched in saturated fatty acid (SFA), and initial data would suggest that SFA enriched high fat diets are particularly potent with respect to reducing pAMPK expression in adipose tissue and bone marrow derived macrophages (BMDM) [2]. Interestingly, recent data suggests that altered AMPK activation is not just a function of obesity but may play a role in energy homeostasis. Chronic activation of AMPK, through a mutation in the γ2 subunit, was involved in hyperphagia, obesity and impaired pancreatic function, which was observed in both mice and humans [18]. Therefore, long-term, non-discriminate activation of AMPK should also be taken with caution. It is possible that the adverse effect of obesity on pAMPK is mediated via adiponectin, the adipokine responsible for regulating glucose levels and fatty acid synthesis, which is decreased in obesity [19]. Adiponectin can activate AMPK via LKB1, but this is impaired in the skeletal muscle of *ob*/*ob* mice [20]. AMPK can be pharmacologically induced through treatment with the agonist, 5′-aminoimidazole-4-carboxamide ribonucleotide (AICAR). Mice given AICAR in conjunction with an HFD showed increased plasma adiponectin levels, reduced macrophage infiltration into the kidney, and reduced kidney hypertrophy, but the impact on adipose tissue biology was not investigated in this study [21].

### 2.2. The Involvement of AMPK in Insulin Resistance

AMPK expression is altered in insulin resistant obese individuals, with reduced expression compared to insulin sensitive body mass index-matched counterparts [15]. AMPKβ1^−/−^ contributes to insulin resistance with decreased phosphorylation of protein kinase B (Akt), increased adipose non-esterified fatty acids, hyperglycemia and hyperinsulinemia [16]. Serum leptin levels increased, and adiponectin reduced, indicating the negative effect of AMPK signaling disruption on adipose biology. Furthermore, in a co-culture system of AMPKα1^−/−^ macrophages and 3T3-L1 adipocytes, insulin stimulated phosphorylation of the insulin receptor and subsequent insulin stimulated glucose uptake, were both decreased with the deletion of the AMPK subunit. This provides confirmation that AMPKs anti-inflammatory effect in macrophages leads to a positive effect on adipose biology [22]. Adipocyte-specific deletion of AMPKβ subunits had a deleterious effect on glucose tolerance and insulin sensitivity, in response to an HFD. Further analysis demonstrated that reduced energy expenditure in brown adipose tissue and hepatic steatosis, but not white adipose tissue inflammation, was responsible for the insulin resistant phenotype [23].

Leclerc and colleagues demonstrated that glucose could regulate and reduce AMPK activity in both human and rodent islets, which resulted in decreased insulin secretion [24]. AMPK can regulate insulin-induced gene expression of L-type pyruvate kinase and pre-proinsulin promotor in islets [25]. A study by Mottillo and colleagues utilized an inducible model for deletion of the two AMPK β subunits in adipocytes (iβ1β2AKO) [26], whereby deletion of AMPK exacerbated the insulin resistant phenotype in terms of hepatic steatosis and glucose tolerance in response to an HFD [23]. AMPK knockout models are associated with differing phenotypes, based on which subunit is targeted. Those with the α1/2 subunit deficiency show increased lipolysis [27], whereas β1/2 deficiency show reduced oxidative metabolism [23]. AMPK activation has been shown to inhibit the first phase of adipogenesis [28] but also increase peroxisome proliferator-activated receptor γ (PPAR-γ) expression [29] in other studies. Despite these confounding results, AMPKα1- and AMPKα2-deficient mice display hypertrophic adiposity following an HFD [27,28,30], but these were conducted in whole body knock-out models. Global AMPKα1^−/−^ mice display increased adiposity, systemic insulin resistance and increased inflammation. When the bone marrow cells were deficient in AMPKα1, the mice had insulin resistance but no obesity, whereas adipocyte AMPKα1 deficiency had the same phenotype as the global knockout [31]. In genetically obese *ob*/*ob* mice, improved insulin sensitivity was a consequence of increased AMPK levels and glucose production in the liver, but no effects were observed in the skeletal muscle of these mice [20]. When AMPK levels were increased with metformin in HFD-rats switched to a chow diet, the metformin group had reduced weight gain and plasma glucose and triglyceride levels compared to a diet switch alone [17]. The authors speculated that metformin was increasing renal FAO by modulating AMPK/acetyl-CoA carboxylase (ACC) pathway and thereby reducing renal lipotoxicity. Metformin treatment in HFD mice also improves glucose metabolism and decreases the adipocyte size compared to HFD fed mice alone [32], thereby illustrating the multitude of effects that AMPK plays in whole body metabolism.

### 2.3. AMPK and Its Link to Cancer

As in obesity, AMPK is downregulated in cancer. AMPK has been implicated in cancer due to its effects on cellular growth and metabolism [33,34]. Tumor cells downregulate AMPK, and thus re-configure cellular metabolism towards glycolytic metabolism to enhance cell growth and proliferation. The mechanistic basis of which is complex and may be related to LKB1 dependent AMPK Thr^172^ phosphorylation to downregulate AMPK-mediated metabolism, over-signalling of the insulin/IGF1-regulated protein kinase Akt/PKB pathway, and/or other mechanisms [35]. LKB, the upstream kinase of AMPK activation, is a tumor suppressor but is mutated in cancer cells. Mammalian target of rapamycin (mTOR) controls cell growth and proliferation and is a target of AMPK. When LKB1 is deficient in cancer cells, mTOR activity inhibition is lost and thus cell proliferation is increased [36]. Cell growth is an ATP-consuming pathway and as such, AMPK directly inhibits mTOR activity. Fatty acid synthesis is increased in many cancers and through AMPKs ability to inhibit this process, AMPK is thought to have anti-cancer roles. Pharmacological AMPK activation, with metformin or salicylate, may protect against cancer initiation and development. Aspirin treated patients with CVD and metformin treated patients with type 2 diabetes have a lower incidence of cancer [37,38]. Whilst these studies seem promising, it is important to note that whether AMPK is a tumor suppressor or potential oncogene, at different cancer stages, is highly controversial and ripe for investigation [35,39,40]. Given the important regulatory role AMPK mediates within the context of metabolism, the next section will deal with the extent to which metabolism may alter inflammation. Also, the impact of nutrient-derived compounds is detailed in Section 4.

## 3. The Intersection between Metabolism and Inflammation

It is now appreciated that there is a complex and dynamic inter-relationship between metabolism and inflammation. Thus, rather than investigating simple pathways/biological processes, there is an intense interest in identifying potential hubs that co-regulate metabolism and inflammation, and in this context, AMPK is an interesting candidate. As stated previously, obesity can affect AMPK activity, but it is difficult to know if the HFD obesogenic effect reflects energy overload or a direct impact of dietary fatty acid (FA) composition. Interestingly, there is an increasing body of evidence that suggests that the nature of fatty acids may co-regulate inflammation and pAMPK expression. Attenuated pAMPK expression is associated with an inverse increase in inflammatory markers in both mouse models of obesity [16] and in obese humans [15]. In nutrient-rich conditions, and with inflammatory stimuli, AMPK phosphorylation and the activity of the α 1 subunit of AMPK is reduced. However, when AMPK is activated there is a subsequent reduction in both nuclear factor kappa B (NF-κB) and tumour necrosis factor α (TNF-α) secretion in macrophages [22].

### 3.1. AMPK and the NLRP3 Inflammasome

The nucleotide-binding domain, leucine-rich-containing family, pyrin domain-containing-3 (NLPR3) inflammasome, mediates interleukin-1β (IL-1β) production, but requires a metabolic product, reactive oxygen species (ROS), for its complete activation. NLRP3 has been shown to play a downstream role in metabolism, with Nlrp3^−/−^ mice on an HFD displaying increased FAO [41]. Caspase-1 is required to process pro-IL-1β to IL-1β activation and *ob*/*ob* mice administered a caspase-1 inhibitor also display increased FAO [42]. AMPK can no longer reduce FAO when the regulatory β-1 subunit is deleted, thereby altering the tetrameric formation of AMPK. This occurs through changes in ACC phosphorylation and altered mitochondrial content [16]. AMPK can inhibit inducible nitric oxide synthase production as a way to reduce inflammation [43], while AMPK inhibition blocks autophagy increasing mitochondrial ROS production, an instrumental step in NLRP3 inflammasome activation [44]. ROS and nicotinamide adenine dinucleotide phosphate inhibition, as well as AMPK activation by AICAR, can all prevent the lipopolysaccharide (LPS)- and palmitic acid (PA)- induction of pro-IL-1β and mature IL-1β production [44]. NLRP3 inflammasome components and the activation of caspase-1 and IL-1β were significantly upregulated in monocyte-derived macrophages from T2D patients and were reduced following treatment with metformin, an insulin sensitizing drug that activates AMPK. The ability of metformin to reduce NLPR3 inflammasome activation was attributed to a reduction in mitochondrial ROS, a known activator of the inflammasome [45].

Metformin has been widely used in the treatment of T2D [3], wherein it induces not only metabolic but also positive anti-inflammatory effects. Metformin indirectly activates AMPK by inhibiting mitochondrial ATP synthesis [46], while decreasing the secretion of inflammatory markers, cyclooxygenase-2 and IL-1β, in the kidney of HFD-fed rats [17]. Citrate is involved in FA synthesis and has also been noted as being a potent inflammatory stimulus [47]. Accumulation of citrate can inhibit phosphofructokinase and back up the glycolytic pathway. The treatment of BMDM with citrate increased NLRP3-dependent IL-1β production and caspase-1 cleavage [48]. The isocitrate/pyruvate cycle has also been implicated in increasing glucose-stimulated insulin secretion, but this area of research warrants further investigation [47]. Treatment of BMDM with α-ketoglutarate has been shown to increase interleukin-10 (IL-10) but also significantly enhance IL-1β expression compared to LPS alone [49]. Conflicting reports however demonstrate that LPS-primed BMDM stimulated with α-ketoglutarate derivative attenuated the LPS-induced IL-1β mRNA and IL-1β protein, through reduced activity of prolyl hydroxylases, which is responsible for stabilising hypoxia-inducible factor 1-α (HIF-1α) protein [50].

### 3.2. Cellular Metabolism and Its Effect on Immune Cell Function

Early work suggested that sub-acute chronic inflammation that typified diet related diseases including obesity, T2D and atherosclerosis disrupted metabolism [51,52,53], wherein cytokines impeded a range of metabolic pathways such as insulin signaling, and reverse cholesterol transport [51,52,53,54,55]. However, it is now evident that there is a much more dynamic reciprocal regulatory relationship between metabolism and inflammation, wherein the nature of cellular metabolism determines immune cells functionality and response. At the simplest level, the cellular balance or switch between oxidative phosphorylation (OxPHOS) versus glucose dependent glycolytic metabolism, defines the functional nature of inactive versus activated immune and proliferating cells. Interestingly immune cells preferentially utilize different metabolic pathways depending on their pro-inflammatory, anti-inflammatory or resolving nature. The metabolic pathway selected reflects the functionality and requirements of the immune cell; whether it is a rapid increase in energy to carry out an innate immune response, or a prolonged availability of energy to enable adaptive responses, such as healing and repair [56]. An elegant combined transcriptomic and metabolomic analysis of BMDM functionality identified important transcriptional and metabolic rewiring during the macrophage polarization [57]. Inactive BMDM rely on the tricarboxylic acid (TCA) cycle and OxPHOS for cellular metabolism, wherein fatty acids and glutamine, but to a lesser extent glucose, are fully oxidized for maximal ATP generation. AMPK plays a key role in modulating metabolism and the balance between OxPHOS and glycolytic metabolism [11]. Upon activation, pro-inflammatory immune cells shift from utilizing the TCA cycle and generation of ATP via OxPHOS to anaerobic glycolysis [57]; this metabolic switch favors the use of glucose as the energy substrate [58]. The pro-inflammatory gram-negative bacterial product, LPS, is often used to induce a classically activated M1 macrophage M1 pro-inflammatory response, as it increases glycolysis and decreases oxygen consumption [50]. This results in a decrease in the AMP:ADP ratio, and a depletion of ATP. When this occurs, there is a shift in ATP production from OxPHOS to glycolysis, as glycolysis produces less ATP. As a result, AMPK would thus be activated to replenish the ATP levels [49]. Increased glycolysis is facilitated by a switch in the phosphofructokinase isoenzymes to its’ ubiquitous isoform, leading to increased phosphorylation of fructose-6-phosphate, the rate limiting step in glycolysis [56].

### 3.3. Metabolic Reprogramming of T Cells

Metabolic reprogramming occurs in T cells, with regulatory T cells favoring OxPHOS and T effector cells switching to glycolysis upon activation. Treg cells have increased AMPK activation and in turn increased lipid oxidation, while T effector cells activate the opposing mTOR pathway [59]. T effector cells have significantly increased glucose transporter 1 (GLUT1) expression compared to T regulatory cells, to deal with the increased need for glycolysis. T regulatory cells require lipid metabolism for their differentiation which is mediated by AMPK where metformin administration to mice increases the number of T reg cells [60]. T effector cells can switch their metabolism under low glucose conditions by decreasing glycolysis and maintaining ATP levels by employing glutamine-dependent OxPHOS [61]. Specifically, LKB1 is critical for multiple T cell functions including development, viability, metabolism and activation. LKB1 was found to be involved in glucose metabolism as T cells lacking LKB1 had increased glycolysis and glucose uptake. A consequence of the disrupted metabolism in LKB1-deficient T cells was an increase in inflammatory CD4^+^ and CD8^+^ cells. In vivo studies in mice lacking T-cell specific AMPKα1 confirmed that AMPK activation was required for these processes [62]. AMPKα1 is required for T effector cells to maintain metabolic flexibility in times of nutrient stress and for T helper cell development in response to infection [61]. When T effector cells need to revert back to metabolically quiescent memory cells, AMPKα1null CD8 T cells were unable to make this transition, further confirming the role that AMPK plays in T cell metabolic switching [63]. Pozanski and colleagues have reviewed T cell and natural kill cell immune-metabolism, and recent emerging evidence suggests that AMPK may also play a role in the metabolic phenotype of natural killer cells [64]. Given the important inter-relationship between metabolism and inflammation, which is partly attributable to AMPK, we now address the extent to which this is sensitive to nutritional status and/or nutritional interventions. Thus, to explore the extent to which AMPK metabolic-inflammation may be either up- or down-regulated by different nutritional components, and how metabolic switching can also lead to beneficial changes in the cell [65] 

## 4. Modulation of AMPK Activation by Nutrients

### 4.1. Fatty Acids Differentially Affect AMPK Function

One of the main drivers of obesity is the excessive consumption of dietary fat. Dietary fat can have widespread negative effects in relation to inflammation, insulin resistance and metabolism, depending on the nature of the fatty acids. Briefly, fatty acids can be either saturated or unsaturated, wherein the latter are sub-divided into monounsaturated (MUFA) or polyunsaturated (PUFA) fatty acids depending on the number of double bonds in the carbon chain. The inclusion of double bonds and the degree of unsaturation has very different effects on metabolism and inflammation. From the experimental point of view, rodent high fat diets are usually enriched with lard which is rich in the saturated fatty acid (SFA) lauric acid (C12:0) or palm oil which is enriched in another SFA palmitic acid (PA) (C16:0). Whilst it has long been acknowledged that obesity is associated with reduced pAMPK expression and a pro-inflammatory phenotype, the true nature of the apparent reciprocal regulation and putative impact of different dietary components is ill defined.

In vitro studies suggest that different fatty acids modulate AMPK mediated inflammation. AMPKβ1^−/−^ BMDM display a predominant M1 profile, which was further exacerbated with two saturated fatty acids, PA and stearic acid (C18:0). Hematopoietic deletion of AMPKβ1^−/−^ was sufficient to induce systemic inflammation in an HFD setting [16]. PA-treated macrophages display increased inflammatory markers, but this is reduced with metformin co-treatment, a phenomenon mimicked in vivo in HFD mice treated with metformin. When AMPK was inhibited with compound C (CC) and included with metformin in vitro, inflammatory markers were increased, thus confirming that metformin is mediating its anti-inflammatory effect through AMPK activation [32]. Interestingly the AMPK activator, AICAR impeded LPS- and FA-induced cytokine response, in part through NF-κB inhibition [16]. However, AICAR has its non-specific limitations, it can inhibit NF-κB DNA binding in human macrophages without AMPK activation [66].

Unsaturated fatty acid may have less of an adverse effect on AMPK mediated metabolism and inflammation (Figure 2). Two groups have compared the impact of palmitic acid and oleic acid (OA) on AMPK. In vitro, direct stimulation with PA reduces pAMPK expression in macrophages. In contrast two MUFA, both palmitoleic acid (PO) (C16:1) and OA (C18:1) do not [2,67]. In vivo, feeding SFA enriched HFD amplifies insulin resistance, inflammation and reduces pAMPK expression. In contrast, feeding an HFD derived from the MUFA, OA did not reduce adipose pAMPK, despite obesity, compared to an SFA enriched HFD [2]. In order to define if the apparent co-regulation of IL-1β inflammation and AMPK was fatty acid dependent, a series of in vitro experiments using CC and AICAR demonstrated that when AMPK was inhibited with CC, IL-1β was increased in BMDM. Conversely, AMPK activation with AICAR in conjunction with PA could inhibit IL-1β secretion [2]. When AMPK is activated pharmacologically through AICAR or by overexpression, it reduces TNF-α- and PA- induced increases in NF-κB expression in endothelial cells [68]. Alpha-linoleic acid (ALA) is n-3 polyunsaturated fatty acid (n-3 PUFA) (C18:3 n-3 *cis*-∆^3^) which acts as a naturally occurring antioxidant and is a cofactor for mitochondrial respiratory enzymes [69]. It is important to note that α- linoleic acid is not a fatty acid per se, but an organosulfur compound derived from the fatty acid caprylic acid (C8:0). ALA was shown to improve both insulin-stimulated glucose uptake and FAO in the skeletal muscle of diabetes prone, obese rats. This was attributed to AMPK activation and reduced lipid accumulation, as dominant-negative AMPK prevented the positive effects of ALA [70]. Subsequent studies found an improvement in whole body glucose tolerance in HFD + ALA rats but did not attribute this to an AMPK mediated lowering of intramuscular lipid [71]. Within the liver of HFD rats, ALA can reduce hepatic lipogenesis through reduced SREBP-1 expression. As a result, hepatic steatosis was attenuated, and this was mediated in part by AMPK activation [72]. ALA can directly increase AMPK in hepatocytes but its lipid lowering and beneficial effects on hepatic metabolism were not AMPK-dependent [73]. In terms of understanding the relative impact of fatty acids versus glucose, Kratz and colleagues identified a distinct population of metabolically activated macrophages (MMe), following a palmitate, glucose and insulin challenge within the adipose tissue. MMe macrophages display attributes of both alternatively activated M2 anti-inflammatory markers (lipid metabolism) but secreted M1-associted pro-inflammatory cytokines [74]. It was noteworthy that PA was more potent in this system compared to glucose and insulin metabolic challenges.

### 4.2. Reversing Metabolic Inflammation through AMPK

The concept of reversing HFD induced metabolic-inflammation is intriguing and involves AMPK. Bone marrow derived macrophages (BMDM) derived from HFD mice retained a “dietary memory” with increased mRNA levels of TNF-α, interleukin-6 (IL-6) and nitric oxide synthase 2, compared to those of a low-fat diet [75], which could be reversed with incubation of the n-6 MUFA, *cis*-PO (C16:1 *cis*-Δ^9^). This PO mediated anti-inflammatory effect was AMPK-dependent. Both OA and PO maintain AMPK expression, and this is thought to mediate their anti-inflammatory effects [75]. Work in Thp-1 human macrophages containing a knockdown of AMPK showed the same inflammatory response by OA when AMPK could not function [2]. Co-incubation of PO with PA, restored pAMPK levels to that of PO alone in BMDM. AMPK inhibition results in PO-induced increases in nitric oxide expression [75]. PA, in a similar manner to the inflammatory stimulus LPS, can increase the cells use of glycolysis in BMDM, while exposure to PO increased OxPHOS. Furthermore, when PO was co-incubated with PA, they displayed similar oxygen consumption levels compared to PO alone. This was thought to reflect the increased M2 phenotype induced through PO incubation [75]. Anti-inflammatory M2 macrophages employ the OxPHOS pathway, which is capable of producing more ATP than glycolysis [57]. M2 macrophages rely on FA as an energy source [58]. The inhibition of various steps of the mitochondrial OxPHOS pathway were able to reduce the expression of arginine 1, a common M2 marker, and abrogate the anti-inflammatory potential of these cells, but had no effect on classically activated macrophages [58]. Activation of pyruvate kinase isozymes M2 enhanced IL-10 secretion in LPS-stimulated BMDM, favouring an M2 phenotype and reduced M1 polarisation [65]. Macrophage M1 polarisation was reduced through AMPK activation by fibronectin type III domain-containing 5 (FNDC5), and worsened when FNDC5 was inhibited, thus lowering AMPK levels in the adipose tissue of HFD-fed mice [76]. Palmitate-treated macrophages treated with AICAR was able to reduce the number of M1 macrophages while subsequently increasing the M2 macrophage population [32]. Microarray analysis of adipose tissue following an 8wk SFA diet showed upregulation of pathways involved in immune function and inflammation. Consumption of a MUFA diet showed no change or downregulation of the same pathways [77]. MUFA-HFD mice display increased energy expenditure compared to SFA-HFD mice, and given the role of AMPK as an energy sensor, the involvement of AMPK in regulating this process cannot be ruled out [2].

### 4.3. AMPK Regulation by Resveratrol, Berberine and Curcumin

Resveratrol, a natural phytochemical, increases glucose uptake in insulin-resistant 3T3-L1 adipocytes by increasing the phosphorylation of pAkt and downstream AMPK activation. A similar effect was noted in vivo in resveratrol treated KKAy mice who demonstrated increased pAkt and elevated AMPK expression [78]. Resveratrol can inhibit cell proliferation in the HT-29 cancer cell line, which was AMPK-dependent. The ability of resveratrol to increase ROS production was elucidated as the mechanism of AMPK activation [79]. Berberine is a naturally occurring plant-derived compound that can indirectly activate AMPK. Berberine can increase energy expenditure and improve liver function in obese mice by stimulating fatty acid oxidation and inhibiting lipogenic genes [80]. Berberine inhibits complex I of the mitochondria to reduce respiration in cultured myotubes and muscle mitochondria. Furthermore, a derivative of berberine demonstrated improved insulin sensitivity and reduced adiposity in vivo in HFD rats [81], owing to the possibility of nutrient intervention treatments. Berberine has positive effects on cancer by inhibiting their metastatic potential and reducing the inflammatory COX-2 pathway by increasing AMPK phosphorylation through increased ROS production [82]. Curcumin is a naturally occurring polyphenol and is the active constituent of turmeric. In a review by Shehzad and colleagues detailing curcumin studies over a 10-year period, this polyphenol was found to have multiple anti-inflammatory and insulin sensitizing effects, involving the suppression of anti-inflammatory transcription factors, upregulation of adiponectin and interactions with glucose and insulin signal transduction pathways [83]. HFD supplemented with 0.15% curcumin reduced body weight, adiposity, serum lipid and glucose levels, and insulin resistance in mice compared to HFD alone controls. Furthermore, beneficial effects on hepatic lipid accumulation and metabolism were noted with reductions in ACC and fatty acid synthase but increased FAO [84]. Curcumin can also increase FAO in muscle which was mediated in part by AMPK and improved palmitate-induced insulin resistance in L6-myotubes [85]. Curcumin demonstrates chemo preventative properties by inducing cell death through p53 phosphorylation, but this is prevented if AMPK action is inhibited with CC treatment in an ovarian cancer cell line [86].

### 4.4. AMPK Regulation by Flavonoids

Plant flavonoids are plant secondary metabolites known to have beneficial health effects [87]. Genistein, a soy isoflavone, can reduce blood glucose in a KK-Ay/Ta Jcl T2D mouse model. When used in vitro in L6 myotubes, genistein stimulates phosphorylation of AMPK and increases glucose uptake by increasing GLUT4 translocation to the plasma membrane, which was prevented when AMPK was inhibited with CC [88]. Genistein has anti-inflammatory properties in macrophages through inhibition of NF-κB mediated by AMPK [89]. In a diabetic rat model (Zucker *fa*/*fa*) fed soy protein, AMPK and FAO were increased in the skeletal muscle and associated with decreased weight gain and improved glucose and triglyceride levels, demonstrating the positive effect that flavonoids can have on overall metabolism [90]. Apigenin, another member of the flavonoid family, dose-dependently increases AMPK expression, while inhibiting adipogenic gene expression and lipolysis in 3T3-L1 adipocytes [91]. In vivo administration of apigenin to HFD-mice reduced the HFD-induced weight gain, increased serum glucose and muscle pro-inflammatory cytokine expression. The apigenin treated mice displayed increased OxPHOS and citrate synthase activity compared to the muscle HFD alone mice, through improvements in mitochondrial biogenesis and mitochondrial function [92]. Similar improvements were observed by Jung and colleagues, with additional benefits observed in relation to improved hepatic steatosis, reduced plasma FFA, and downregulation of lipogenic genes in the liver of apigenin + HFD mice [93]. Flavonoids have been ascribed cancer preventative roles through the induction of apoptotic pathways mediated through AMPK signaling pathways [94]. The ability of FA to undergo metabolic reconfiguration, combined with the anti-oxidant and anti-inflammatory properties of nutrients and how these are involved in the diet-induced modulation of inflammation therefore requires further investigation. FA are an important source of metabolites and no doubt their metabolism will feed more into the immuno-metabolism circuit, however to date the nature and extent to which dietary FA and/or nutritional status can modulate this dynamic two-way process requires definition.

## 5. Conclusions and Future Perspectives

AMPK is an exciting target given its co-regulatory role with respect to metabolism and inflammation. It is evident that Western dietary elements, which are obesogenic in nature, attenuate AMPK, the metabolic impact of which probably explains the sub-acute pro-inflammatory phenotype that typifies obesity, T2D and CVD. The challenge remains to discern if/how certain nutrients either up- or down-regulate cellular AMPK status. It is evident that feeding SFA are particularly deleterious, and therefore negatively impact upon metabolism and sub-acute chronic inflammation that typifies obesity T2D. Speculatively it is probable that high-fructose diets will also have deleterious effect on AMPK, given the fact that feeding fructose increases endogenous synthesis of SFA. Conversely, there is some evidence to suggest that feeding high fat diets rich in MUFA and PUFA, or supplemented with natural AMPK agonists, such as resveratrol and berberine, do not attenuate AMPK. Furthermore, sirtuin 1 (SIRT) is another nutrient sensor that has widespread effects on metabolism in response to caloric restriction and may also be involved in regulating metabolism and inflammation [95]. Whilst the paradigm of nutritional enhancement of AMPK activity is worthy of investigation in light of the obesity epidemic, to date the impact of these nutritional interventions have been observed in mice. Therefore, we need to define if/how this translates to humans.

## Abbreviations

ACC	Acetyl-CoA carboxylase
ADP	Adenosine diphosphate
AICAR	5′-aminoimidazole-4-carboxamide ribonucleotide
Akt	Protein kinase B
ALA	A-linoleic acid
AMP	Adenosine monophosphate
AMPK	AMP-Activated Protein Kinase
AMPKK	AMPK kinase
ATP	Adenosine triphosphate
BMDM	Bone marrow derived macrophages
CaMKK	Ca^2+^/calmodulin-dependent protein kinase kinases
CC	Compound C
CVD	Cardiovascular disease
FA	Fatty acid
FAO	Fatty acid oxidation
FBP	Fructose-1,6-bisphosphate
FNDC5	Fibronectin type III domain-containing 5
Glut1	Glucose transporter 1
HIF-1α	Hypoxia-inducible factor 1-α
HFD	High fat diet
IL-1β	Interleukin-1β
IL-6	Interleukin 6
IL-10	Interleukin 10
LKB1	Liver kinase B1
LPS	Lipopolysaccharide
Mme	Metabolically activated macrophages
mTOR	Mammalian target of rapamycin
MUFA	Monounsaturated fatty acids
OA	Oleic acid
OxPHOS	Oxidative phosphorylation
P	Phosphorylation
PA	Palmitic acid
PO	Palmitoleic acid
PPAR-γ	Peroxisome proliferator-activated receptor γ
PUFA	Polyunsaturated fatty acid
ROS	Reactive oxygen species
SAT	Subcutaneous adipose tissue
SFA	Saturated fatty acid
SIRT1	Sirtuin
SREBP-1	Sterol regulatory element-binding protein 1
T2D	Type 2 diabetes
TCA	Tricarboxylic acid
Thr	Threonine
TNF-α	Tumour necrosis factor α
VAT	Visceral adipose tissue
α	Alpha
β	Beta
γ	Gamma
